# Tryptophan-Catabolizing Enzymes – Party of Three

**DOI:** 10.3389/fimmu.2014.00485

**Published:** 2014-10-09

**Authors:** Helen J. Ball, Felicita F. Jusof, Supun M. Bakmiwewa, Nicholas H. Hunt, Hajime J. Yuasa

**Affiliations:** ^1^Molecular Immunopathology Unit, School of Medical Sciences and Bosch Institute, University of Sydney, Sydney, NSW, Australia; ^2^Department of Physiology, Faculty of Medicine, University of Malaya, Kuala Lumpur, Malaysia; ^3^Laboratory of Biochemistry, Faculty of Science, Department of Applied Science, National University Corporation Kochi University, Kochi, Japan

**Keywords:** convergent evolution, divergent evolution, indoleamine 2,3-dioxygenase, tryptophan 2,3-dioxygenase, gene duplication, immunoregulation

## Abstract

Indoleamine 2,3-dioxygenase (IDO) and tryptophan 2,3-dioxygenase (TDO) are tryptophan-degrading enzymes that have independently evolved to catalyze the first step in tryptophan catabolism via the kynurenine pathway (KP). The depletion of tryptophan and formation of KP metabolites modulates the activity of the mammalian immune, reproductive, and central nervous systems. IDO and TDO enzymes can have overlapping or distinct functions depending on their expression patterns. The expression of TDO and IDO enzymes in mammals differs not only by tissue/cellular localization but also by their induction by distinct stimuli. To add to the complexity, these genes also have undergone duplications in some organisms leading to multiple isoforms of IDO or TDO. For example, many vertebrates, including all mammals, have acquired two IDO genes via gene duplication, although the IDO1-like gene has been lost in some lower vertebrate lineages. Gene duplications can allow the homologs to diverge and acquire different properties to the original gene. There is evidence for IDO enzymes having differing enzymatic characteristics, signaling properties, and biological functions. This review analyzes the evolutionary convergence of IDO and TDO enzymes as tryptophan-catabolizing enzymes and the divergent evolution of IDO homologs to generate an enzyme family with diverse characteristics not possessed by TDO enzymes, with an emphasis on the immune system.

## Evolution of Tryptophan-Catabolizing Enzymes

**Figure 1:**
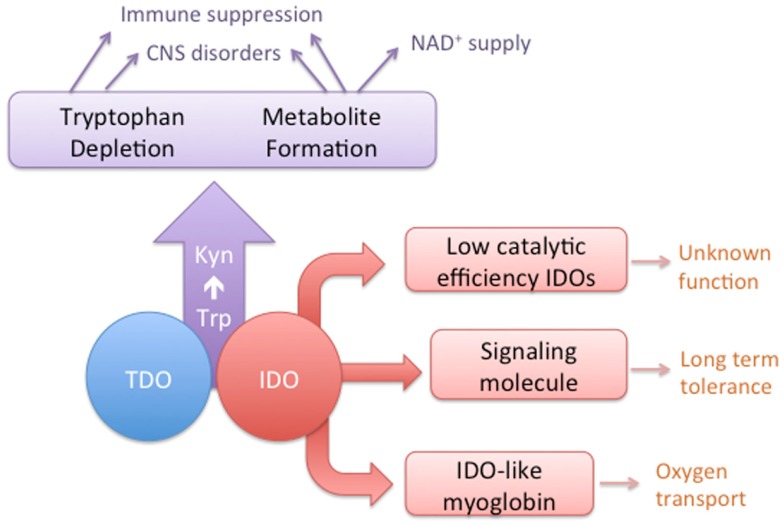
**A conceptual diagram depicting the overlapping activities of IDO and TDO enzymes and the distinct characteristics of some IDO homologs**.

The concepts of convergent and divergent evolution can apply to multi-cellular organisms or at the level of gene families, such as those that encode a particular enzymatic activity [reviewed in Ref. (1)]. In the first case, distinct enzyme superfamilies can evolve to catalyze the same reaction, i.e., functional convergence. Second, sequence divergence within families of enzymes can lead to functional divergence of enzyme homologs. In this review, we bring together and analyze evidence, mostly gathered in the last decade, which shows that the enzymes catalyzing the first step in the kynurenine pathway (KP) are a fascinating example of both these processes (Figure 1). The two enzymes, tryptophan 2,3-dioxygenase (TDO) and indoleamine 2,3-dioxygnease (IDO), are structurally distinct proteins that nonetheless have evolved to catalyze the same reaction, the conversion of tryptophan (Trp) to *N*-formylkynurenine. The result of TDO and IDO activity is the depletion of Trp and production of metabolites of the KP. For mammals, Trp is an essential amino acid with most dietary Trp being metabolized through the KP (2). Some Trp is also required for synthesis of protein and the neurochemical serotonin. Activity of the KP can affect the levels of tryptophan, thereby modulating serotonin synthesis or causing suppression of cell proliferation. In addition, the production of metabolites can provide a source of nicotinamide dinucleotide (NAD^+^) and have other biological effects, particularly in the immune, reproductive, and central nervous systems. Depending on their expression pattern, TDO and IDO enzymes may have similar or distinct biological activities. Furthermore, in some organisms, gene duplication has resulted in homologs of either TDO or IDO. In the case of IDO homologs, there is evidence of functional divergence that has evolved in the case of one of the duplicated genes.

### Evolution of TDO

**Figure 2:**
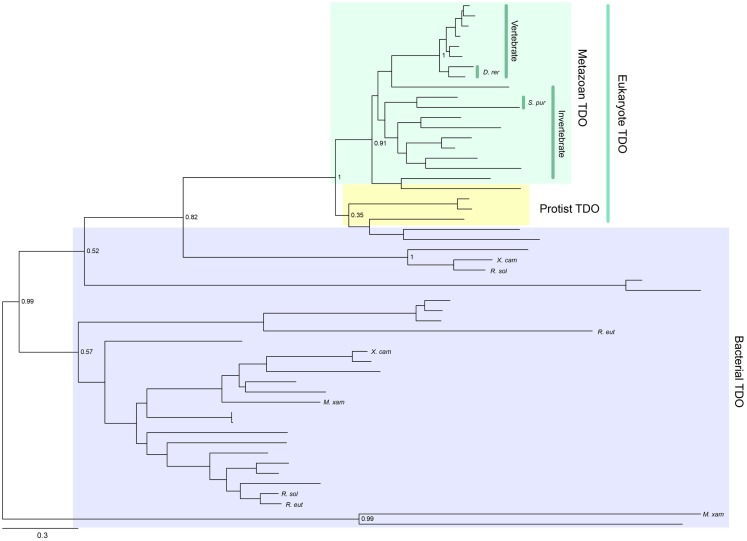
**Phylogenetic relationships of known TDOs constructed with the maximum-likelihood method (unrooted tree)**. Multiple sequence alignment at the amino acid level was generated using the MUSCLE program (3) and the ML tree was constructed using MEGA 6 (4). The internal branch labels are bootstrap values with 100 replications. *D. rerio* (*D. rer*) and *S. purpuratus* (*S. pur*) have two TDOs. A few bacteria, *Myxococcus xanthus* (*M. xan*), *Ralstonia solanacearum* GMI1000 (*R. sal*), *R. eutropha* JMP134 (*R. eut*), and *Xanthomonas campestris* 33913 (*X. cam*), also have two TDOs. A complete tree, with names of all species, is shown in Figure S1 in Supplementary Material.

Tryptophan 2,3-dioxygenase is widely distributed across species, from metazoans to bacteria, but has not been found in fungi. Its enzymatic activity has been conserved consistently throughout metazoan evolution (Yuasa and Ball, manuscript submitted). A phylogenetic tree showing the distribution of TDO enzymes in metazoan species is presented in Figure 2. Gene duplications have resulted in some organisms possessing two TDO genes, e.g., *Danio rerio* (zebrafish) and *Stongylocentrotus purpuratus* (sea urchin). TDO is found in several hundred species/strains of bacteria. They do not form a monophyletic group, and some bacterial TDOs show sequence homology with eukaryotic TDOs. A few bacteria have two TDO genes; however, they are located distant from each other in the phylogenetic tree (Figure 2). This suggests that multiple horizontal gene transfer events have occurred in bacterial TDO evolution.

### Evolution of IDO

**Figure 3:**
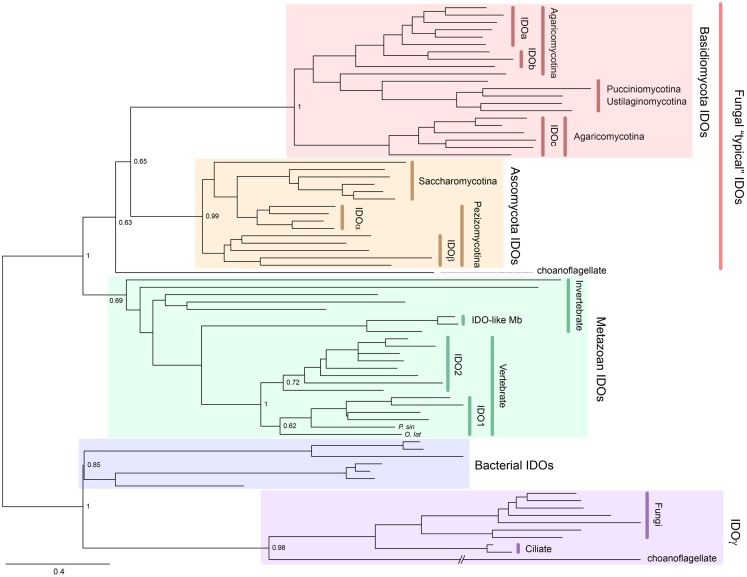
**Phylogenetic relationships of known IDOs and IDO-related proteins constructed with the maximum-likelihood method (unrooted tree)**. Multiple sequence alignment at the amino acid level was generated using the MUSCLE program (3) and the ML tree was constructed using MEGA 6 (4). The internal branch labels are bootstrap values with 100 replications. The Medaka-fish, *Oryzias latipes* (*O. lat*) and a soft-shelled turtle, *Pelodiscus sinensis* (*P. sin*) have a putative IDO1. A complete tree, with names of all species, is shown in Figure S2 in Supplementary Material.

Indoleamine 2,3-dioxygenase is also widely distributed from bacteria to metazoans and, unlike TDO, can be found in fungi. Gene duplication has occurred in a number of lineages to generate IDO homologs. For example, some Ascomycota fungi possess *IDO*α, *IDO*β, and *IDO*γ, while some Basidiomycota fungi have *IDOa, IDOb*, and *IDOc* (5–7). A phylogenetic tree showing the distribution of IDO enzymes is presented in Figure 3. Mammals also possess more than one IDO enzyme, namely, IDO1 and the more recently discovered IDO2 (8–10). Examining the sequence divergence of the IDO1 and IDO2 proteins would suggest a gene duplication event before the origin of the tetrapods (8). However, the presence of two IDO homologs in mammals, but only one IDO2-like enzyme in lower vertebrates, suggests that the ancestral *IDO2* gene was more recently duplicated, before the rise of the mammals (10). Recently, IDO homologs, one with higher homology to mammalian IDO1 compared to IDO2, have been detected in a species of fish and turtle (Yuasa et al., manuscript in preparation). We speculate that this indicates that the duplication event was more ancient and that the *IDO1* gene has been lost in a number of lower vertebrate lineages.

## Functional Convergence of IDO and TDO

**Figure 4:**
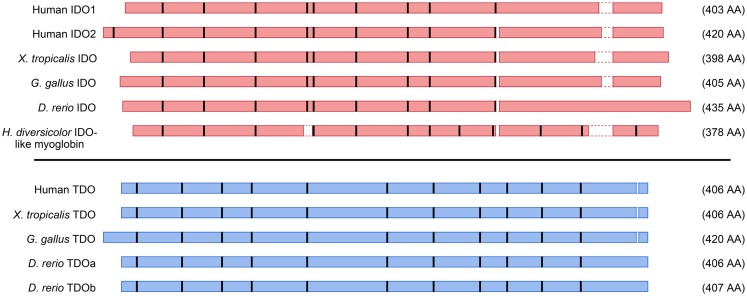
**Schematic representation of amino acid alignments for selected IDO and TDO proteins**. Amino acid contigs are shown as red (IDO) or blue (TDO) boxes, and positions of introns indicated by thick vertical bars. Gaps less than two amino acids in the alignments were omitted. Selected sequences are from human beings, frog (*Xenopus tropicalis*), chicken (*Gallus gallus*), zebrafish (*Danio rerio*), and abalone (*Haliotis diversicolor*).

The genomic structures and sequences of the *IDO* and *TDO* genes do not suggest a common ancestor, rather that the two genes have evolved separately to catalyze the same reaction. Figure 4 depicts amino acid homologies and genomic structures for some selected TDO and IDO proteins and their homologs. Both enzymes are heme-containing proteins and the crystal structures of human IDO1 and bacterial TDOs have been obtained (11–13). IDO1 is a monomeric enzyme while TDO is tetrameric with one heme per monomer. Although there is low sequence identity between the enzymes, the crystal structures reveal similarities in the heme-binding environment and substrate-binding site (13). The step-by-step process by which Trp is converted to *N*-formylkynurenine by heme dioxygenases has been investigated over many years, and the reaction mechanism is thought to be broadly similar between the two enzymes (14).

Structurally-unrelated enzymes that catalyze the same reactions have been termed analogous (rather than homologous) enzymes. Examples of analogous enzymes are found in many classes of enzymes, but are particularly clustered in those involved in synthesis and hydrolysis of polysaccharides, effects of oxygen on cell components, and synthesis/turnover of cell walls (15). A suggested mechanism for the evolution of analogous enzymes is that a changed substrate specificity or reaction mechanism results in the recruitment of an enzyme to perform a new role (15). One difference between mammalian TDO and IDO enzymes is the substrate selectivity; TDO is selective for the l-Trp enantiomer, whereas IDO has a broader substrate range of indole-containing compounds. IDO1 was originally isolated from rabbit intestine as a d-Trp-catabolizing enzyme (16) and its substrates include d- and l-Trp, tryptamine, 5-hydroxytryptophan, and 5-hydroxytryptamine (17). The biological significance of the oxidation of other substrates by IDO1 is not clear and the substrate range of ancestral IDO enzymes is also unknown. We hypothesize that ancestral IDO enzymes were broadly indole-oxidizing enzymes and mammalian IDO1 has evolved into an enzyme with high affinity and efficiency for l-Trp as a substrate, with this activity forming the basis of its biological actions.

It has been observed that analogous enzymes are more likely to have a skewed phylogenetic distribution; for example, one isoform evolves in archaebacteria while the other is present in both metazoan and bacteria (1). The absence of TDO enzymes in fungi suggests that IDO enzymes are responsible for Trp metabolism in this kingdom. In bacteria, some species possess TDO or IDO enzymes and they occur exclusively to each other, suggesting that only one is required for Trp metabolism (18). However, many organisms, including all mammals, possess both TDO and IDO and there is the potential for the enzymes to have overlapping or distinct actions, depending upon their expression patterns. For example, mammalian TDO is expressed in the liver and its activity is upregulated by glucocorticoids and l-Trp (19, 20). IDO1 has constitutive expression in some tissues, such as the lung, but its expression can be widely induced by certain stimuli that are significant in immunity and inflammation, including interferon-γ (IFNγ), lipopolysaccharide (LPS), and tumor necrosis factor (TNF) (21–23). IDO2 has constitutive expression in the liver (8) and can be induced by cytokines in certain cell types (9, 24, 25).

The following sections discuss some examples of IDO and TDO having either overlapping or distinct biological effects based on their common enzymatic activity and distinct expression patterns, with a general focus on pathways and networks that are significant in immunity and/or inflammation. The interactions of KP metabolites with the aryl hydrocarbon receptor (AhR) are described in more detail in other articles in this issue. The roles of Trp-catabolizing enzymes in the placenta and central nervous system also have been reviewed recently (26, 27).

### Immune evasion by tumors

Expression of Trp-catabolizing enzymes, whether in cancer cells or dendritic cells in tumor-draining lymph nodes, has been associated with suppression of the effector T-cell responses toward tumors and, hence, with a poorer prognosis. A number of pathways are implicated [reviewed in Ref. (28)], including suppression of T-cell proliferation through sensing of Trp depletion via the mTOR and/or GCN2 kinase pathways; generation of Treg cells via either the GCN2 kinase pathway and/or kynurenine (Kyn) activating the AhR; and the pro-apoptotic properties of some KP metabolites on T-cells. IDO1 expression, associated with these effects, has been found in plasmacytoid dendritic cells (29) and human tumors (30). Human IDO2 mRNA expression has been detected in gastric, colon, and renal tumors (24) and mRNA/protein in myeloid and plasmacytoid dendritic cells (31). TDO activity in gliomas has been linked to activation of the AhR and reduced antitumor immune responses (32). TDO expression also has been found in a significant proportion of human tumors, and a TDO inhibitor restored the ability of mice to reject TDO-expressing tumors (33). While the relative importance of tumor versus dendritic cell expression is debatable, it is clear that there is the potential for both IDO and TDO enzymes to be involved in the suppression of immune responses toward tumors. Selective inhibitors can distinguish the role of TDO and IDO enzymes, but it is more difficult to define the role of IDO1 versus IDO2 using pharmacological approaches despite some recent progress in that direction (34). There is some confusion regarding the selectivity and mode of action of the enantiomers of the most commonly used IDO inhibitor, 1-methyl-tryptophan [reviewed in Ref. (35)], so genetic approaches are also helpful for defining the function of these enzymes; for example, an IDO inhibitor was ineffective at preventing tolerance of transplanted melanoma in *Ido1*^−/−^ mice, suggesting that the inhibitor acted via IDO1 (36). These *Ido1*^−/−^ mice recently have been found to express an enzymatically inactive form of IDO2 in specific immune cell types (37), which should be considered when drawing conclusions. However, silencing the *Ido1* gene by siRNA resulted in reduced tumor growth in B16F10 tumor-bearing mice (38). In addition, *Ido2^−/−^* mice did not reproduce the reduced susceptibility to inflammatory skin cancer seen in the *Ido1*^−/−^ mice (37). IDO2 activity may have a role in promoting Treg generation (discussed in Treg Cells and Tolerance); however, it appears that IDO1 is the homolog predominantly associated with immune evasion by tumors. Selective inhibitors of TDO or IDO enzymes have been proposed as adjunctive chemotherapies. The IDO inhibitor 1-methyl-tryptophan can also act as Trp mimetic, signaling amino acid sufficiency in the mTOR pathway (39). This action has the potential to relieve some of the immune suppression caused by all three enzymes, thus modulation of downstream pathways may provide broader efficacy as an adjunctive therapy than would selectively targeting Trp-catabolizing enzymes.

Thus, the evidence for IDO1 modulating host–tumor cell interactions is strong and diverse, but involvement of TDO and IDO2 is much less studied and requires further corroboration.

### Treg cells and tolerance

The importance of the activation of the AhR by kynurenines in the development of tolerance is reviewed in Ref. (40) and other articles in this issue. Critical to the development of tolerance is the generation and maintenance of Treg cells, with the kynurenine-mediated activation of the AhR playing an important role (41). As previously mentioned, TDO-mediated activation of the AhR has been linked to reduced immune responses toward gliomas (32). In addition, *Tdo^−/−^* mice had greater inflammatory responses and mortality after LPS administration, similar to *AhR^−/−^* mice (42). This suggests that the AhR is activated by kynurenines formed by TDO in certain situations. The longer term development of immune tolerance may depend on sustained kynurenine production in particular microenvironments, such as dendritic cells. Mice develop a tolerance to LPS, as they show reduced inflammatory responses and mortality on re-exposure. This tolerance was observed to be dependent on the combined presence of IDO1 and the AhR, suggesting that IDO1 is important for the longer term modulation of the immune response to LPS (42). This study demonstrated a positive feedback loop where signaling through the AhR induced IDO1 expression, which in turn produced sustained activation of the AhR. This is similar to the transforming growth factor β (TGF β)/IDO1 axis, in which a positive feedback loop results in sustained expression of IDO1, TGFβ, type I interferons, and the generation of Treg cells [reviewed in Ref. (40)]. Interestingly, the establishment of this axis is associated with a non-enzymatic function of IDO1 (see Signaling Properties of IDO1) (43). Kynurenine administration failed to restore tolerance to LPS administration in *Ido1^−/−^* mice, suggesting that non-enzymatic capabilities of IDO1 may also play a role in establishing tolerance to endotoxins (42).

Many studies have identified IDO1 as the Trp-catabolizing enzyme predominantly involved in the generation of Treg cells, although we emphasize that the pharmacological approaches employed in those studies would not distinguish between IDO1 and IDO2. For example, IDO inhibition with 1-methyl tryptophan was shown to reduce Treg formation by CpG oligonucleotide-stimulated human dendritic cells and in the lungs of *Aspergillus fumigatus*-infected mice (44, 45). Other studies utilize the *Ido1^−/−^* mouse; however, use of this strain is complicated by cell-specific alternative-splicing leading to some loss of IDO2 activity (37). Two studies employing both *Ido1^−/−^* and *Ido2^−/−^* mouse strains showed that the formation of Treg cells either may be modulated by IDO2 (37) or is specific to IDO1 activity (42), depending on the model. In addition, siRNA knockdown of either IDO1 or IDO2 in human dendritic cells reduced the formation of kynurenine and Treg cells (31). The tolerance to LPS administration was specific to IDO1 expression, as IDO2 deletion had no effects on outcomes in either initial or subsequent administrations of LPS (42). The generation of Treg cells in mice treated with CpG oligonucleotide has been defined as IDO1 mediated (46), but the generation of Treg cells was also reduced in *Ido2^−/−^* mice in this model (37).

In summary, all three Trp-catabolizing enzymes have the ability to suppress immune responses. However, the longer term maintenance of the Treg balance may require sustained expression in particular microenvironments. This appears to occur in some TDO- or IDO-expressing tumors and IDO-expressing dendritic cells. It is noteworthy that IDO2 expression was found to be constitutive in human myeloid and plasmacytoid dendritic cells, while IDO1 showed a more restricted expression pattern, dependent on prostaglandin E_2_ (31). Overall, we conclude that the different expression patterns of the IDO proteins may determine whether each is tolerogenic in response to specific stimuli.

### NAD^+^ supply

NAD^+^ is an essential co-factor required by many biochemical processes. It is formed either by a *de novo* synthesis pathway, from metabolites of the KP, or via a salvage pathway using nicotinic acid (NA) and nicotinamide (vitamin B3). Thus, a yeast strain with its only Trp-catabolizing gene (*BNA2*, an IDO homolog) deleted becomes a NA auxotroph. Expressing IDO/TDO enzymes in this mutant strain will rescue the yeast if the enzyme has sufficient Trp-catabolizing activity to provide a source of NAD^+^. Many IDO enzymes do rescue the mutant strain, including mouse and human IDO1 enzymes, IDOα/β enzymes from Pezizomycotina, and IDOa/b enzymes from Agaricomycotina (7). As fungi do not possess TDO, it is likely that IDO activity has a role in supplying NAD^+^ in these microorganisms. NAD^+^ synthesis is not only determined by the activity of the Trp-catabolizing enzymes but also the presence of all the downstream enzymes. One of these enzymes, quinolinic acid phosphoribosyl-transferase, has been found to be active only in the liver and kidney of rodents (47, 48). It has been shown that the KP is the major route of NAD^+^ supply in rat hepatocytes (49). Two Trp-catabolizing enzymes are constitutively expressed in mouse liver – TDO and IDO2 (8, 19). TDO is most likely to be involved in NAD^+^ supply since TDO enzymes, but not IDO2 enzymes, were able to rescue the NA-auxotrophic yeast strain [Yuasa and Ball, manuscript submitted (7)]. The lack of significant activity of critical downstream enzymes for *de novo* synthesis would suggest that most extrahepatic tissues rely on the salvage pathway for NAD^+^ supply. Nevertheless, *in vitro* studies on human brain cells have suggested that the KP pathway can contribute to maintaining NAD^+^ and perhaps the distribution and activity of the enzymes in the pathway has not been fully elucidated (50). In addition, while *Tdo*^−/−^ mice showed significantly reduced levels of circulating NAD^+^ when placed on a NA-deficient diet, compared to a complete diet, they still maintained optimal rates of growth (48). The amount of NAD^+^ in the liver was consistent in wildtype and *Tdo*^−/−^ mice fed either the complete or NA-deficient diets. Measurements of metabolites in the urine showed that the conversion rate of l-Trp to Kyn was increased in the *Tdo*^−/−^ mice on the NA-deficient diets. This suggests that extrahepatic IDO1 enzyme activity was increasing the circulating pool of Kyn, which could then be further metabolized in the liver to synthesize NAD^+^ for recirculation to extrahepatic tissues. We may speculate that IDO2 activity in the liver also contributes to maintaining the levels of NAD^+^ in that tissue, in the absence of both NA in the diet and TDO activity.

NAD^+^ supply also has modulatory effects in cancer and inflammation. Inhibition of NAD^+^ formation has been proposed as a chemotherapeutic target due to high rates of NAD^+^ consumption by tumor cells. This is suggested to be predominantly due to increased ADP-ribosylation from polyADP-ribose polymerase activity (51). Although an inhibitor (FK866) of an enzyme in the salvage pathway is efficacious as a chemotherapeutic agent (52), little is known of the contribution of the *de novo* synthesis pathway to tumor cell metabolism. NAD^+^ is also critical for mediating the effects of sirtuins, some of which modulate inflammatory pathways. Again, the relative contributions of the *de novo* synthesis pathway and the salvage pathways in sirtuin activation are unclear, although an IDO inhibitor was shown to reduce both NAD^+^ levels and sirtuin 1 activity in human primary astrocytes (53). It should be noted that sirtuin 1 activity has been associated with reduced Foxp3 stability and suppression of Treg cell formation (54), in contrast to the Treg-promoting effects of the KP. In summary, while NAD^+^ supply regulates tumor cell metabolism and inflammatory responses, the roles of the two supply pathways, as well as each of the enzymes within them, still require elucidation. We suggest that a focus on the KP–NAD^+^ axis in tumor and immune cells would provide valuable information both to clarify certain anomalies in the field and to determine whether it is possible to interfere with this pathway in tumor cells without compromising immune cell functions.

## Divergent Evolution of l-Trp-Catabolizing Enzymes

TDO and IDO have evolved to have similar enzymatic functions, and these enzymes coexist in most animals. In addition, gene duplications have resulted in some species having more than one homolog of each enzyme. For example, zebrafish have one IDO enzyme and two TDO enzymes. The TDO enzymes are highly conserved throughout vertebrate evolution (Table 1) and zebrafish TDO paralogs have similar enzymatic activity and expression patterns (Jusof et al., manuscript in preparation). *IDO* genes have been duplicated independently in a number of lineages, for example, *IDO1* and *IDO2* in mammals and *IDOa, IDOb*, and *IDOc* in Agaricomycotina fungi. Compared with TDO enzymes, there is lower sequence homology between both IDO paralogs and orthologs (Table 1). The different biochemical and functional properties observed among IDO enzymes is likely a result of the greater sequence divergence. The following sections describe three features found in certain IDO enzymes that are not shared among all IDO enzymes.

**Table 1:** **Amino acid identity over the aligned segments of IDO and TDO enzymes using *blastp* (http://blast.ncbi.nlm.nih.gov)**.

	Human IDO1 (%)	Human IDO2 (%)	*X. tropicalis* IDO (%)	*G. gallus* IDO (%)	*D. rerio* IDO (%)	*H. diversicolor* IDO-like Mb (%)
Human IDO1	100	44	43	44	45	34
Human IDO2	44	100	53	60	50	37
*X. tropicalis* IDO	43	53	100	55	50	36
*G. gallus* IDO	44	60	55	100	55	38
*D. rerio* IDO	45	50	50	55	100	36
*H. diversicolor* IDO-like Mb	34	37	36	38	36	100

	**Human TDO (%)**	***X. tropicalis* TDO (%)**	***G. gallus* TDO (%)**		***D. rerio* TDOa (%)**	***D. rerio* TDOb (%)**

Human TDO	100	82	84		75	75
*X. tropicalis* TDO	82	100	86		77	78
*G. gallus* TDO	84	86	100		77	76
*D. rerio* TDOa	75	77	77		100	76
*D. rerio* TDOb	75	78	76		76	100

*No significant similarity (*E* value < 1) was observed when aligning the TDO proteins with IDO proteins. Selected sequences are from human beings, frogs (*Xenopus tropicalis*), chickens (*Gallus gallus*), zebrafish (*Danio rerio*), and abalone (*Haliotis diversicolor*)*.

### Low catalytic-efficiency IDO enzymes

IDO enzymatic activity requires the reduction of the heme iron from its ferric form to its ferrous form. Characterization of IDO activity often begins by assessing the activity of the protein in a reaction containing methylene blue (MB) as an electron source, among other constituents including the substrate (55). IDO1 enzymes are highly efficient at metabolizing Trp in the MB assay. In contrast, IDO2 enzymes have a much higher *K*_m_ and lower *V*_max_ for Trp in this reaction (10, 56–58). The physiological reductant of IDO enzymes originally was proposed to be superoxide anion (59) and more recently suggested to be cytochrome *b*_5_ (60, 61). Human IDO1 showed reduced activity in a reaction containing cytochrome *b*_5_ compared with the MB assay (61). In contrast, the Trp-catalyzing efficiency of mouse IDO2 was greatly improved in the cytochrome *b*_5_ reaction, although it was still significantly less efficient than mouse IDO1 (56). The dependence of enzymatic activity on co-factors underscores the difficulty in assessing the biochemical characteristics of IDO homologs. It is certainly possible that the optimal reaction system to observe IDO2 activity has not been developed. Despite this limitation, several other lines of evidence suggest that IDO2 enzymes have lower Trp-metabolizing activity than the IDO1 counterparts. First, the amount of Kyn formed in mammalian cells transfected with mouse IDO2 is lower than in cells transfected with mouse IDO1 (8, 9). In addition, human IDO2 expression produced significantly less Kyn than mouse IDO2 (9, 62). Second, expression of IDO1, but not mouse or human IDO2, rescued a NA-auxotrophic yeast strain (7). This suggests that IDO2 enzymes do not possess sufficient activity to supply the metabolites required for NAD^+^ supply in yeast. On the other hand, knockdown of human IDO2 expression in dendritic cells by siRNA significantly suppressed Kyn formation and this was associated with decreased generation of Treg cells (31). Thus, IDO2 may have sufficient enzymatic activity to generate a biological effect in certain systems. The constitutive expression of IDO2 in subsets of dendritic cells, compared with the regulated expression of IDO1, may be an example where the weak enzymatic activity of IDO2 is still able to produce a distinct biological effect, compared with IDO1, by virtue of its unique expression pattern (31).

*Ido2*^−/−^ mice, while having unaltered plasma Kyn levels, show reduced skin contact hypersensitivity responses and attenuated Treg cell generation (37). In addition, *Ido2*^−/−^ mice have selectively less autoantibody production, compared with overall antibody production, leading to reduced joint inflammation in a spontaneous model of arthritis (63). Interestingly, the effect was unrelated to serum Kyn levels, because these were unchanged in *Ido2* ^−/−^ mice while *Ido1* ^−/−^ mice had reduced circulating Kyn but no attenuation of joint inflammation. Furthermore, while 1-methyl-tryptophan could inhibit Kyn formation in IDO2-expressing HEK293T cells, it did not affect IDO2-mediated suppression of T-cell proliferation in co-culture experiments (62). Taken together, these findings may point to some effects of IDO2 being mediated via a mechanism unrelated to Trp metabolism/Kyn formation, although, in the mouse model, it is possible that localized enzymatic activity is sufficient to produce the effect.

Interestingly, other lineages outside the metazoan kingdom have independently generated IDO homologs through gene duplication events. Some of these IDO homologs have very low catalytic activities, similar to IDO2 enzymes, as tested in the MB assay (5, 6, 18). The low enzymatic activity of these homologs was confirmed by the lack of rescue of the NA-auxotrophic yeast strain (7). These low catalytic-efficiency enzymes include IDOγ in Perzizomycotina and IDOc in Basidiomycota. The role of the low catalytic-efficiency IDOs in Trp metabolism, or another metabolic process, is not well understood. l-Trp is metabolized by both IDO1 and IDO2 enzymes; however, assessing tryptophan derivatives has revealed substrates, such as 5-methoxytryptophan, which are metabolized by human IDO2, but not human IDO1 (58). Human IDO2 has a higher affinity for some of these substrates than for l-Trp. Additionally, we found that Trp metabolism by mouse IDO2 could be inhibited by a much wider range of compounds compared with mouse IDO1 (34). We conclude that IDO2 enzymes interact with a different, but overlapping, range of substrates/inhibitors compared to IDO1 enzymes.

Recent studies show that IDO1 enzymes can possess heme peroxidase and indole peroxygenase activity in the presence of hydrogen peroxide and relevant substrates (64, 65). These activities have yet to be investigated in other IDO enzymes’ paralogs/orthologs. The biological significance of these alternative reactions is unknown. However, as IDO enzymes with relatively low efficiency at converting Trp to Kyn have been maintained in several lineages during evolution, we believe that it would be of interest to investigate whether these enzymes catalyze these alternative reactions more efficiently than the conversion of Trp to *N*-formylkynurenine.

Lymphocytes appeared early in vertebrate evolution, with the emergence of jawed fish, and the adaptive immune system has become increasingly complex as vertebrates have evolved through to mammals. Another feature of mammalian evolution is the development of the chorion into the larger, more complex placenta. We speculate that the development of these two systems has favored acquisition of high Trp-catabolizing efficiency in IDO1 enzymes in order to fulfill particular biological roles. Fungi do not have TDO and it may be that high Trp-catabolizing efficiency IDO enzymes, IDOα/β and IDOa/b, have evolved in fungi to provide a source of NAD^+^. The occurrence and conservation of IDO enzymes with lower catalytic activity for Trp metabolism in different kingdoms suggests that these enzymes are still fulfilling a role, albeit one that is less well understood. We conjecture that potential roles may include Trp metabolism in particular microenvironments, or metabolism of other substrates including through an alternative oxidation reaction.

### Signaling properties of IDO1

It was shown that IDO1 expression, but not IDO1 catalytic activity, was necessary for the immunoregulatory effects leading to longer term self-tolerance of plasmacytoid dendritic cells treated with TGF-β (43). Immunoreceptor tyrosine-based inhibitory motifs (ITIMs) were phosphorylated in mouse IDO1 in response to TGF-β. This was followed by upregulation and recruitment of tyrosine phosphatases, SHP-1 and SHP-2, and the initiation of a cascade of downstream events favoring activation of the non-canonical NF-κB pathway, leading to the sustained expression of IDO1, TGF-β, and interferon-α. Two ITIMs (ITIM1 and ITIM2) were observed to be present in an alignment of human being, rat, dog, and mouse IDO1 proteins. In contrast, a tyrosine to phenylalanine substitution in IDO2 proteins meant that only ITIM2 was present. The presence of only one ITIM was shown to prevent the recruitment of the phosphatase, so that mouse IDO2 does not possess the same signaling capability as mouse IDO1. In addition, the ITIM motifs were demonstrated to regulate the SOCS3-dependent degradation of the IDO1 protein in response to other cytokines, thus possessing both these motifs also provides an additional mechanism for regulating IDO1 protein turnover (66). The expression of human IDO2 in dendritic cells was found to be SOCS3-independent, correlating with its lack of two ITIM motifs (31). Thus, sequence differences in the IDO1 and IDO2 proteins have resulted in an additional signaling function and mechanism of regulation for one of the homologs.

There is some evidence that IDO2 acts through effector pathways independent of enzymatic activity. As discussed earlier, the attenuation in joint inflammation in *Ido2*^−/−^ mice was unrelated to circulating Kyn levels and an inhibitor of IDO2-catalyzed Kyn formation did not relieve IDO2-mediated suppression of T-cell proliferation (62, 63). Trp depletion induces translation of the liver inhibitory protein via the GCN2 kinase pathway, and this could be reversed by Trp supplementation in IDO1-expressing cells but not in IDO2-expressing cells (9). We suggest that these findings imply that potential alternative signaling properties of IDO2 should be investigated.

### IDO-like myglobin

Hemoglobins and myoglobins from bacteria, plants, and animals are thought to have evolved from a common ancestral gene encoding a 14–16 kDa protein [reviewed in Ref. (67)]. A myoglobin isolated from the abalone *H. diversicolor*, however, was a 39 kDa protein quite different to previous myoglobin proteins and with significant homology to the IDO family of enzymes (68). The IDO reaction mechanism involves the formation of an oxygenated intermediate with an absorption spectrum similar to that of oxymyoglobin (69). IDO enzymes do not act as oxygen carriers as this intermediate is unstable. The IDO-like myoglobin possibly may have evolved to reversibly bind oxygen through amino acid changes in the heme-binding cavity (67). IDO-like myoglobins are found in several families in the Vetigastropoda and, interestingly, the conventional myoglobin is not present in these families (67).

Thus, in an example of functional convergence with the myoglobin family, the IDO-like myoglobins have evolved to fulfill the function of an oxygen carrier where conventional myoglobins have been lost. However, as an example of functional divergence, IDO-like myoglobins have lost the ability to metabolize tryptophan and to function as an IDO enzyme (Yuasa and Ball, manuscript in preparation).

## Summary

Convergent evolution usually occurs by two different proteins evolving to fill the same biological role in different organisms. For example, TDO is likely to be the enzyme mostly responsible for Trp metabolism leading to NAD^+^ supply in mammals; however, its absence in fungi suggests that IDO enzymes perform this function in fungi. However, both TDO and IDO enzymes, with Trp-catabolizing activity, are present in vertebrates. The functional evolution of IDO proteins is more complex than TDO evolution as efficient Trp-catabolizing activity is not uniformly conserved. Mammalian IDO1s have evolved to be highly efficient at catabolizing Trp. It is possible that the increasing complexity of the vertebrate immune and reproductive systems might have led to selective pressures favoring the acquisition of Trp-catabolizing activity, in particular tissues or situations. Although Trp catabolism, by either IDO or TDO, may have similar effects in some cell types, e.g., tumors, Trp catabolism in dendritic cells that drives tolerance may be an IDO-mediated effect.

Gene duplication is a driving force in evolution as it allows one gene to perform its original function while another, under less selective pressure, may diverge and develop new characteristics and/or lose its original ones. Both *IDO* and *TDO* genes have undergone duplications in some lineages, although IDO proteins show much greater sequence divergence than TDO proteins. This has led to greater diversity within the IDO family, with differing enzymatic activities and signaling capabilities. For example, IDO1 enzymes, but not IDO2 enzymes, possess two motifs that confer a signaling role on the proteins, regulating both expression and stability. Thus, the sequence diversity among IDO proteins leads to a distinct mechanism for sustaining IDO1 expression in dendritic cells and the development of tolerance, in response to specific stimuli. The diverse characteristics and different expression patterns of the Trp-catabolizing enzymes equate to distinct biological roles for the enzymes.

## Conflict of Interest Statement

The authors declare that the research was conducted in the absence of any commercial or financial relationships that could be construed as a potential conflict of interest.

## Supplementary Material

The Supplementary Material for this article can be found online at http://www.frontiersin.org/Journal/10.3389/fimmu.2014.00485/abstract
