# Supplementary material for: Tryptophan-Catabolizing Enzymes – Party of Three
Source: Front Immunol. 2014 Oct 9;5:485. doi: 10.3389/fimmu.2014.00485 (PMC4191572; doi:10.3389/fimmu.2014.00485)
Supplement: Supplementary file 1 [file 111961_Image_1.PDF]

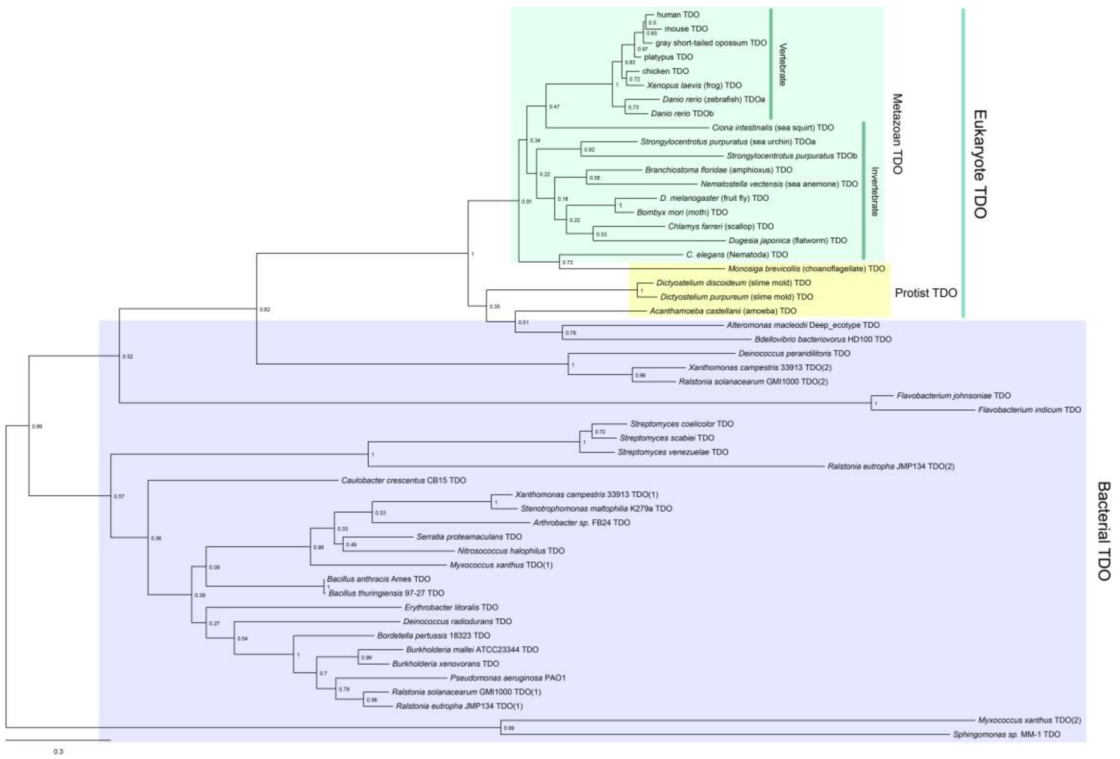

Supplementary Figure 1. Phylogenetic relationships of known TDOs constructed with the maximum-likelihood method (unrooted tree). Multiple sequence alignment at the amino acid level was generated using the MUSCLE program (Edgar, 2004) and the ML tree was constructed using MEGA 6 (Tamura et al., 2013). The internal branch labels are bootstrap values with 100 replications. *D. rerio* (*D. rer*) and *S. purpuratus* (*S. pur*) have two TDOs. A few bacteria, *Myxococcus xanthus* (*M. xan*), *Ralstonia solanacearum* GMI1000 (*R. sal*), *R. eutropha* JMP134 (*R. eut*), and *Xanthomonas campestris* 33913 (*X. cam*) also have two TDOs.
