# Supplementary material for: Tryptophan-Catabolizing Enzymes – Party of Three
Source: Front Immunol. 2014 Oct 9;5:485. doi: 10.3389/fimmu.2014.00485 (PMC4191572; doi:10.3389/fimmu.2014.00485)
Supplement: Supplementary file 2 [file 111961_Image_2.PDF]

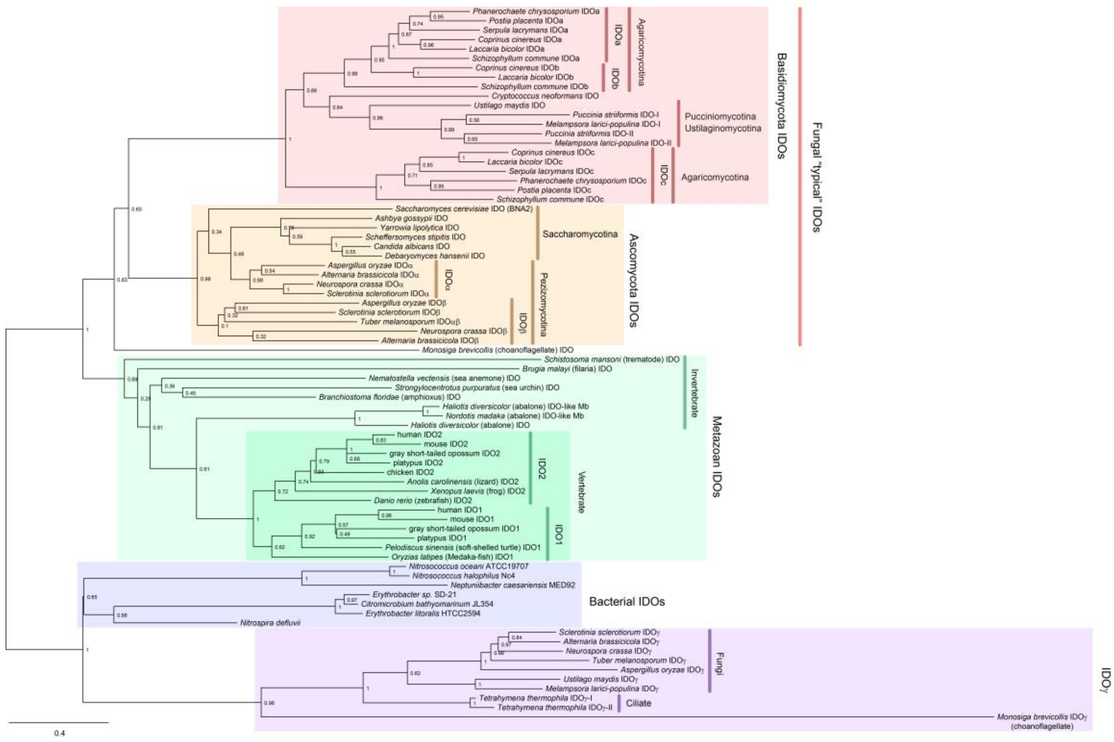

Supplementary Figure 2. Phylogenetic relationships of known IDOs and IDO-related proteins constructed with the maximum-likelihood method (unrooted tree). Multiple sequence alignment at the amino acid level was generated using the MUSCLE program (Edgar, 2004) and the ML tree was constructed using MEGA 6 (Tamura et al., 2013). The internal branch labels are bootstrap values with 100 replications. The Medaka-fish, *Oryzias latipes* (*O. lat*) and a soft-shelled turtle, *Pelodiscus sinensis* (*P. sin*) have a putative IDO1.
